# The Opioid Crisis in America: Too much, too little, too late

**DOI:** 10.5811/westjem.2018.2.38087

**Published:** 2018-03-13

**Authors:** Kristi L. Koenig

**Affiliations:** County of San Diego, Health & Human Services Agency, Emergency Medical Services, San Diego, California, University of California, Irvine, Department of Emergency Medicine, Orange, California

## TOO MUCH

There is widespread awareness of one component of today’s opioid crisis in America – the overuse of opioid medications. With overdose deaths reaching epidemic levels, some U.S. states have issued emergency declarations to bring legal authorities to bear for this unprecedented situation. Following a 2015 fall in life expectancy for the first time in decades, the Centers for Disease Control and Prevention identified opioid overdoses as a major contributor to this increase in population mortality. On October 26, 2017, the President of the United States declared the opioid epidemic to be a “national public health emergency”;^1^ the declaration was renewed on January 24, 2018.

Prescription opioids have been a major contributor to addiction and overdose deaths. While in the not-too- distant past, physicians were trained to treat pain aggressively, and even to consider pain to be a fifth vital sign that must be immediately addressed, strong caution is now advised when prescribing opioids. Comprehensive mitigation strategies have been enacted, including a requirement to check databases of prior opioid prescriptions before dispensing new pain medications.

As the emergency medical services (EMS) medical director for a large county with a population of approximately 3.3 million residents spread over more than 4,000 square miles, I have joined others in the implementation of various strategies to prevent opioid overdose deaths. This includes developing policies and protocols to authorize and train law enforcement and emergency medical technician first responders, in addition to higher-trained personnel such as paramedics, to administer naloxone to patients with hypoventilation after opioid use. But naloxone is a short-lived emergency intervention, not a complete solution to a long-term problem.

## TOO LITTLE, TOO LATE: ANOTHER OPIOID CRISIS

Despite the abundance of opioids in our communities, particularly when compared with other countries, there are patients who legitimately need treatment of their pain and are in danger of not receiving it. Pain should be treated as early as possible to halt its escalation. This is especially true in emergency settings, including treatment by paramedics in the prehospital environment.

Today we are experiencing a national shortage of critical life-saving medications and drugs needed immediately to mitigate suffering.^2^ This “too little” gap has been exacerbated by the recent hurricane event in Puerto Rico – a very important source of medical drug and device manufacturing, which has markedly diminished on account of destruction wrought by the storm, and the glacial pace of recovery and restoration. Should there be a national effort to restore pharma production in the U.S. territory of Puerto Rico? Or an effort to rebuild elsewhere? Or should we expand our efforts to purchase medications from other countries?

This emergency drug shortage crisis – including opioids – has led to challenges in reliable and consistent access to important medications in our nation’s emergency departments and hospitals as well as the prehospital setting. If not addressed more consistently nationwide, could this escalate to the point where we regularly lack the resources to treat pain and other time-sensitive conditions in an emergency situation?

As an EMS medical director, part of my job is to authorize destruction of expired opioids in the prehospital setting. This requirement is tragic, especially when science tells us that these drugs are effective long after their official expiration dates and prehospital agencies are severely challenged by lack of timely access to these suffering-reducing medications. While it is possible to apply for “shelf life extension” and “emergency use authorization” for these expired products,^3^ obtaining authorization is generally not feasible or timely due to the current complex regulatory framework for use of expired drugs. Thus, once the expiration date arrives, it is “too late.”

Important initiatives such as Executive Order 13588, Reducing Prescription Drug Shortages, signed by President Obama on October 31, 2011, and Title X of the Food and Drug Administration Safety and Innovation Act of 2012, signed into law on July 7, 2012, have increased industry notification requirements for impending shortages, but more is needed. An evidence-based, federal extension of authorization for use of expired medications for a reasonable period of time during a period of national shortages might be one option for addressing this emerging new twist on the “too little, too late” national opioid shortage.

## NATIONAL CALL TO ACTION

Critical drug shortages have been addressed in the past, for example, by the Association of State and Territorial Health Officials (ASTHO) in 2012,^4^ with supporting evidence from an Institute of Medicine report entitled “Crisis Standards of Care–A Systems Framework for Catastrophic Disaster Response.”^5^ Yet, this important suite of suggested solutions has not been implemented to any large degree and, in fact, seems to have been dwarfed by the current attention focusing on opioid overdoses.

At the time of publication of the ASTHO document, it was estimated that nearly 40% of the short-supply drugs contributed negatively to emergency care delivery. The report described a menu of strategies to address resource shortfalls, including techniques for conservation, substitution, and adaptation. It further suggested the potential to tap into existing federal and state emergency stockpiles... but the reality is that regulatory authority is generally lacking for this action.

We must rekindle our national efforts to address this other manifestation of the current opioid crisis, that is, the one of “too little, too late.” These emergency drug shortages require critical attention and acknowledgment. Certainly it is essential to limit opioid use when unnecessary as well as to explore non-opioid alternatives for pain treatment. This could include techniques as simple as using ice packs and splinting, as novel as emergency acupuncture, or usage of other less-commonly employed analgesics in the emergency setting such as ketamine, intravenous acetaminophen or ketorolac, and nitrous oxide. The bottom line, however, is that there is a legitimate need for opioids, when properly prescribed.

The emergency drug shortage situation appears to be escalating across America. We need help on the front lines to ensure we will have the means to alleviate suffering from acutely painful conditions. This means exploring the creative solutions mentioned above as well as other innovative, science-supported approaches to provide timely access to analgesia. Certainly let’s put a stop to the declared national opioid crises, but let’s also enact long-term strategies to ensure sufficient opioid production and access for essential patient care. Opioids are an important tool in the armamentarium for pain treatment. While we apply temporary regional mitigation strategies to address critical drug shortages, a long-term solution that identifies and eliminates the root causes of the crisis must be mobilized. In the meantime, our attention should not be solely focused on the popularized opioid crisis. In the case of opioids while there is too much, we also have too little, too late.
